# Relationship Between Vaccination Status and Biopsychosocial Characteristics in Sheltered Adolescents

**DOI:** 10.7759/cureus.12575

**Published:** 2021-01-08

**Authors:** Cecilia E Zemanek, Nina Liu, Ceyda H Sablak, Brittney A Gaudet, Taylor L Jarvill, Allison N Kayne, Jeffrey M Downen, Hope Kincaid, Amy B Smith, Robert D Barraco, Hoonani M Cuadrado, Marna R Greenberg

**Affiliations:** 1 Emergency and Hospital Medicine, University of South Florida Morsani College of Medicine/Lehigh Valley Health Network Campus, Allentown, USA; 2 Network Office of Research and Innovation, University of South Florida Morsani College of Medicine/Lehigh Valley Health Network Campus, Allentown, USA; 3 Division of Education, University of South Florida Morsani College of Medicine/Lehigh Valley Health Network Campus, Allentown, USA; 4 Surgery, University of South Florida Morsani College of Medicine/Lehigh Valley Health Network Campus, Allentown, USA; 5 Community Health and Health Studies, University of South Florida Morsani College of Medicine/Lehigh Valley Health Network Campus, Allentown, USA

**Keywords:** homeless youth, immunization, vaccination, adolescent, demography, homeless persons, sex characteristics

## Abstract

Introduction

Homeless youth are a vulnerable population. A volunteer clinic supported by medical students in northeastern Pennsylvania provides shelter and healthcare to adolescents seeking refuge. We set out to determine the immunization status of youth in the shelter and evaluate for associations of immunization deficiency with demographics or biopsychosocial factors.

Methods

After IRB approval, a retrospective cohort study was performed from existing clinical records at the shelter 2/2015-9/2019. Chart abstraction included variables such as demographics (including age, sex, and race/ethnicity), biopsychosocial factors (including childhood trauma/abuse history, substance abuse history, and sexual activity), and immunization history.

Results

A total of 440 charts were analyzed. When comparing demographics of patients that had complete vaccine regimens versus those who did not, the race was statistically significant (p=.006). The most prominent difference in race was seen for Black/African American patients; only 19.57% had a completed vaccine regimen documented. Regarding immunization history, vaccine schedules of hepatitis B, measles mumps rubella (MMR), inactivated polio vaccine (IPV), and varicella were most likely to be complete; pneumococcal conjugate vaccine (PCV) 13, rotavirus, influenza, and human papillomavirus vaccine (HPV) were least likely. There was no association found between a completed vaccine regimen and biopsychosocial variables. A larger portion of females (37.35%) completed the HPV vaccine compared to males (23.14%) (p=.009).

Conclusions

In this single-site study, this vulnerable, at-risk population of sheltered adolescents lacked the vaccinations recommended by the Centers for Disease Control and Prevention. Racial disparities further compounded this vulnerability for Black/African American teens. Additionally, a significantly greater number of female patients received the HPV vaccine compared to males.

## Introduction

Every year, approximately 1.6 million young Americans face housing insecurity [1]. Homeless adolescents experience disparate health outcomes, with a higher risk for exposure to communicable diseases and higher rates of psychiatric illness, substance use, sexually transmitted infections, and childhood abuse and trauma than their housed peers [2,3]. Despite these disparate health outcomes, homeless adolescents routinely lack access to proper medical care, particularly to receiving recommended vaccinations [4]. As such, there has been a recent effort to improve access to vaccinations; specifically, seeking to obtain immunization history and identify barriers to delivering vaccinations in high-risk settings have become of particular significance to improve access for this vulnerable population [5]. Studies of disparities within this population have been limited, particularly about race, ethnicity, and sexual orientation.

A volunteer shelter-based medical clinic supported by medical students in northeastern Pennsylvania was established in 2015 to ensure that vulnerable, abused, and homeless young people under the care in the region had access to appropriate medical care and necessary counseling services. Since the clinic’s establishment, no demographic assessments or vaccine status reports had been created to elucidate the health deficiencies and biopsychosocial characteristics of the patient population. It was presumed that patients at the shelter likely lacked health-critical vaccinations, however, it had not previously been determined which vaccines were deficient. We set out to determine the immunization status of adolescent youth in the shelter. We additionally hypothesized that specific demographic factors, such as race and sex, and biopsychosocial factors, such as drug use and sexual history, would be associated with deficiencies in vaccine status among this vulnerable population.

This work was accepted for presentation, in part, as an abstract titled “Immunization Status of Homeless Adolescents” at Pennsylvania College of Emergency Physicians, April 2, 2020. Because of restrictions from COVID-19, the abstract was posted online but not presented.

## Materials and methods

A cross-sectional study was performed from existing clinical records of state mandated physicals and acute care visits performed on patients at a youth shelter in Bethlehem, PA between February 19, 2015, and September 05, 2019. The adolescents at this shelter typically spend two weeks in transitional housing until they can be placed in a more stable environment. The shelter may extend that stay as long as six weeks under special circumstances. Included in the analysis were records from those patients who had received state-mandated physical examinations and/or who had received acute care at the shelter. The medical assessments provided at this facility were conducted by a single hospital network. 

Chart abstraction included variables such as demographics, biopsychosocial factors (including childhood trauma/abuse history, substance abuse history, and sexual activity), and immunization history. Immunization history was abstracted from data in the chart provided by school vaccination records while biopsychosocial factors were by patient or parent self-report. All data extracted from the charts was placed in a de-identified database accessible only to members of the study team. The study was reviewed and approved as a minimal risk study by the Lehigh Valley Health Network Institutional Review Board.

Statistical analysis

Descriptive statistics were calculated to summarize the demographic characteristics and baseline medical, behavioral, social, and sexual history for the study sample. The mean with the standard deviation was reported for normally distributed continuous variables. If any of the continuous variables were non-normally distributed, the median was presented with the inter-quartile range (IQR). Categorical variables were presented as frequencies and percentages.

To test the study hypothesis that specific demographic and biopsychosocial factors were associated with vaccine regimen completion status, a chi-square test of Independence was conducted. However, if > 20% of the expected cell counts were < 5, Fisher’s exact test was used instead. For continuous variables, the Independent Samples t-test was used if the distribution of age was normal in each group. If it was not, the Mann-Whitney U test was used instead. Vaccine regimen completion status was a dummy composite variable created with the following variables: hepatitis B, diphtheria, tetanus, pertussis (DTap), Haemophilus influenzae type B (Hib), inactivated polio vaccine (IPV), measles mumps rubella (MMR), and varicella. To be considered complete, all six vaccines must have been received at the proper dose. Those with missing data for any vaccines were not included in this analysis. All statistical analyses were conducted using Statistical Analysis System (SAS) version 9.4 (SAS Institute, Inc., Cary, NC). Analyses were two-tailed and a p-value < 0.05 was considered statistically significant.

## Results

In total, 440 charts were found to fit the inclusion criteria and were reviewed for vaccine and biopsychosocial data. Regarding the demographics of the study population, the majority of patients were female (n=245, 55.68%) and White (n=71, 47.02%) with a mean age of 15.32 (SD=1.62). It should be noted that only 151 of the 440 had data regarding race. Eighty-eight patients reported their ethnicity (Hispanic or Latino or “not” Hispanic or Latino), of which the majority were Hispanic/Latino (n=70, 79.55%). Of the 440 charts reviewed, only 293 (66.59%) patients had a physical record of their immunization history, of which only 109 (37.20%) had a completed vaccine regimen (Table 1).

**Table 1:** Demographics and Immunization Completion. Hep B: hepatitis B; DTaP: diphtheria, tetanus, pertussis; Hib: Haemophilus influenzae type B; IPV: inactivated polio vaccine; MMR: measles mumps rubella; EMR: electronic medical record Note: The number included (N) for each of the variables is presented in the top row of the table for each column.  However, race and ethnicity had missing data so the N’s for those variables are presented on the top row of that section for each column. ^a^110 records were missing all vaccine data and another 37 were missing all vaccine data except for influenza.  All 147 are excluded from the second column and subsequently the third and fourth columns as well. ^b^The vaccine regimen was considered complete if the appropriate number of doses of each of the following six vaccines had been received – Hep B, DTaP, Hib, IPV, MMR, and Varicella.  If any of these vaccines were not given at the correct dosage or were missing from the EMR the vaccine regimen was considered incomplete for that specific record. ^c^P-value is for comparison between those who completed the vaccine regimen and those who did not. The Independent Samples t-test was computed for age; Fisher’s exact test was computed for gender, race, and religious exemption; and the chi-square test was computed for ethnicity. P-values in bold are considered statistically significant. ^d^289 records were missing data for the race overall. ^e^352 records were missing data for ethnicity overall

Demographics					
	Entire Sample (N=440)	Those w/No Missing Vaccine Data^ a^ (N=293)	Completed Vaccine Regimen^b^ (N=109)	Did Not Complete Vaccine Regimen^b^ (N=184)	P-Value^c^
Age mean (SD)	15.32 (1.62)	15.18 (1.53)	15.09 (1.60)	15.23 (1.49)	0.4443
Gender n (%)					0.1047
Male	187 (42.50)	121 (41.30)	38 (34.86)	83 (45.11)	
Female	245 (55.68)	166 (56.66)	70 (64.22)	96 (52.17)	
Transgender	8 (1.82)	6 (2.05)	1 (0.92)	5 (2.72)	
Race^d^ n (%)	(N=151)	(N=106)	(N=46)	(N=60)	0.0057
Black/African American	55 (36.42)	38 (35.85)	9 (19.57)	29 (48.33)	
White	71 (47.02)	52 (49.06)	27 (58.70)	25 (41.67)	
Asian	3 (1.99)	1 (0.94)	0	1 (1.67)	
Hispanic/Latino	0	0	0	0	
American Indian/Alaskan Native	0	0	0	0	
Other	2 (1.32)	2 (1.89)	1 (2.17)	1 (1.67)	
Multiracial	20 (13.25)	13 (12.26)	9 (19.57)	4 (6.67)	
Ethnicity^e^ n (%)	(N=88)	(N=60)	(N=35)	(N=25)	0.3679
Hispanic/Latino	70 (79.55)	47 (78.33)	26 (74.29)	21 (84.00)	
Not Hispanic/Latino	18 (20.45)	13 (21.67)	9 (25.71)	4 (16.00)	
Religious Exemption n (%)					0.5312
Yes	2 (0.45)	2 (0.68)	0	2 (1.09)	
No	438 (99.55)	291 (99.32)	109 (100)	182 (98.91)	

**Figure 1:**
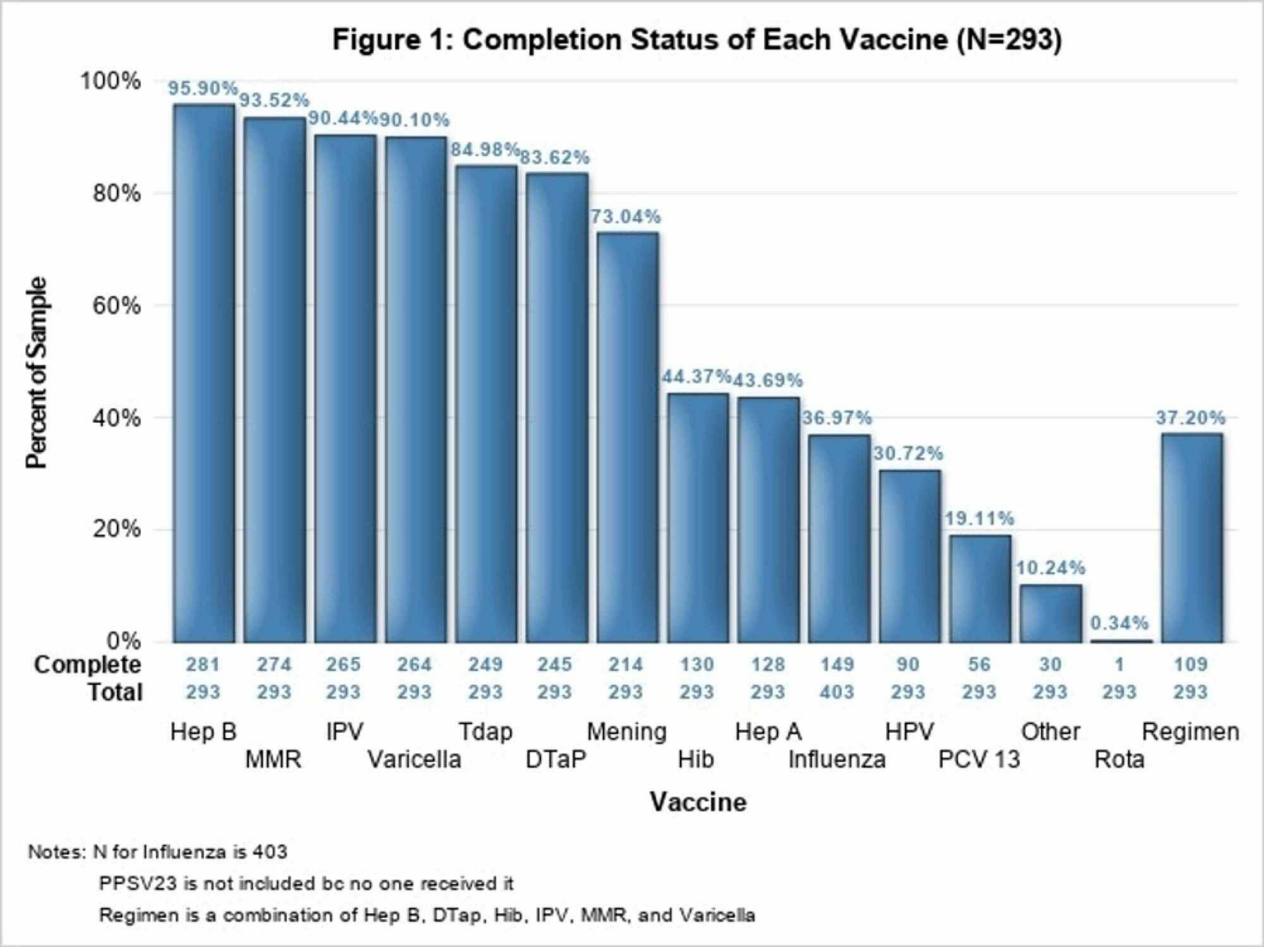
Completion Status of Each Vaccine (N=293).

In terms of immunization history, the highest percentage of patients (n=281, 95.90%) had received all required doses of the hepatitis B vaccine, followed by the MMR vaccine (n=274, 93.52%), the IPV vaccine (n=265, 90.44%), and the varicella vaccine (n=264, 90.10%) (Figure 1). In contrast, only 44.37% of patients had completed all required doses for the Hib vaccine (n=130). Other notable vaccination rates include the hepatitis A vaccine (n=128, 43.69%), influenza vaccine (n=149, 36.97%), and human papillomavirus vaccine (HPV) (n= 90, 30.72%) (Figure 1). Looking at the demographics of those patients who had complete vaccine regimens versus those who did not, the race was found to be statistically significant (p=.006). The most prominent difference in race was seen for Black/African American patients in which only 19.57% had a completed vaccine regimen documented.

Over half of our patients had a documented psychiatric condition (n= 239, 54.94%). A large portion had a history of aggression (n=254, 58.53%) and a history of suicidal/self-injurious attempt (n= 194, 44.80%). The most common drug used was marijuana (n=163, 37.56%), followed by tobacco (n=120, 27.65%), and alcohol (n=52, 12.01%). Two hundred thirty-three (53.69%) of patients were sexually active, with a median first age of sexual activity of 14 years (IQR=13-15). No statistically significant association was found between any of the aforementioned biopsychosocial variables and whether a patient had a completed vaccine regimen (Table 2).

**Table 2:** Biopsychosocial Variables and Immunization Status. IQR: inter-quartile range; Hep B: hepatitis B; DTaP: diphtheria, tetanus, pertussis; Hib: Haemophilus influenzae type B; IPV: inactivated polio vaccine; MMR: measles mumps rubella; EMR: electronic medical record Note: The number included (N) for each of the Medical History variables is presented in the top row of that section for each column. For all other variables (i.e., the Behavioral History, Social History, and Sexual History variables) the N for the entire sample (first column) is the first number in parentheses after the variable name and the N for those with no missing vaccine data (second column) is the second number in parentheses after the variable name. ^a^110 records were missing all vaccine data and another 37 were missing all vaccine data except for influenza. All 147 are excluded from the second column and subsequently the third and fourth columns as well. ^b^The vaccine regimen was considered complete if the appropriate number of doses of each of the following six vaccines had been received – Hep B, DTaP, Hib, IPV, MMR, and varicella. If any of these vaccines were not given at the correct dosage or were missing from the EMR the vaccine regimen was considered incomplete for that specific record. ^c^P-value is for comparison between those who completed the vaccine regimen and those who did not. The chi-square test was computed for all variables except for cardiac (Fisher’s exact test), immune (Fisher’s exact test), and age sexually active (Mann-Whitney U test). None of the p-values were statistically significant. ^d^Combination of suicide six months and suicide past. ^e^Combination of aggressive behaviors six months and aggressive behaviors past.

Biopsychosocial Factors					
	Entire Sample (N=440)	Those w/ No Missing Vaccine Data^ a^ (N=293)	Completed Vaccine Regimen^b^ (N=109)	Did Not Complete Vaccine Regimen^b^ (N=184)	P-Value^c^
Medical History n (%)	(N=435)	(N=289)	(N=108)	(N=181)	
Cardiac	20 (4.60)	11 (3.81)	6 (5.56)	5 (2.76)	0.3403
Pulmonary	99 (22.76)	68 (23.53)	26 (24.07)	42 (23.20)	0.8661
Immune	2 (0.46)	2 (0.69)	1 (0.93)	1 (0.55)	1.0000
Other (non-psychiatric)	171 (39.31)	103 (35.64)	40 (37.04)	63 (34.81)	0.7017
Psychiatric	239 (54.94)	155 (53.63)	55 (50.93)	100 (55.25)	0.4759
Behavioral History n (%)					
Suicide 6 month (N=434, 288)	97 (22.35)	59 (20.49)	23 (21.30)	36 (20.00)	0.7919
Suicide past (N=432, 287)	170 (39.35)	101 (35.19)	41 (37.96)	60 (33.52)	0.4451
Suicide ever^d^ (N=433, 288)	194 (44.80)	117 (40.63)	43 (39.81)	74 (41.11)	0.8283
Aggressive 6 month (N=434, 288)	156 (35.94)	107 (37.15)	43 (39.81)	64 (35.56)	0.4690
Aggressive past (N=433, 288)	226 (52.19)	153 (53.13)	58 (53.70)	95 (52.78)	0.8788
Aggressive ever^e^ (N=434, 288)	254 (58.53)	169 (58.68)	66 (61.11)	103 (57.22)	0.5164
Traumatic Event (N=434, 288)	114 (26.27)	76 (26.39)	29 (26.85)	47 (26.11)	0.8902
Social History n (%)					
Tobacco (N= 434, 288)	120 (27.65)	75 (26.04)	29 (26.85)	46 (25.56)	0.8083
Alcohol (N= 433, 287)	52 (12.01)	35 (12.20)	13 (12.15)	22 (12.22)	0.9855
Marijuana (N= 434, 288)	163 (37.56)	110 (38.19)	42 (38.89)	68 (37.78)	0.8510
Other Drug (N= 434, 288)	42 (9.68)	28 (9.72)	10 (9.26)	18 (10.00)	0.8372
Sexual History n (%)					
Sexually Active (N= 434, 288)	233 (53.69)	149 (51.74)	58 (54.21)	91 (50.28)	0.5190
Age Sexually Active median (IQR) (N=224, 145)	14 (13-15)	14 (13-15)	14 (13-15)	14 (13-15)	0.8866
Birth Control (N= 422, 278)	155 (36.73)	98 (35.25)	39 (36.45)	59 (34.50)	0.7411
STI (N= 427, 283)	27 (6.32)	17 (6.01)	7 (6.54)	10 (5.68)	0.7677

An additional analysis was conducted to further understand if sexual activity and gender were associated with HPV vaccine deficiencies. There was no statistical significance in HPV deficiencies when looking at whether the patient was sexually active; 30.87% of those who were sexually active completed the HPV vaccine compared to 30.22% of those who were not sexually active (p=.904). However, when looking at gender, there was a statistically significant difference; a larger portion of females (37.35%) completed the HPV vaccine as compared to males (23.14%) (p=.009).

## Discussion

This single-site study confirmed that many adolescents at a shelter lack the vaccinations recommended by the Centers for Disease Control and Prevention (CDC). Only 37.20% of our patients, with complete data, had received the required doses for the hepatitis B, DTap, Hib, IPV, MMR, and varicella vaccines. Many of our patients were missing multiple vaccines or had not received all of the required doses of a vaccine. Homeless individuals are at increased risk for sexually transmitted diseases and conditions like hepatitis A and hepatitis C, so it is concerning that our patients had relatively low rates of vaccine completion for those respective conditions [6,7]. In addition, only about a third of our patients had received a flu shot, even though influenza is a common medical condition seen among homeless individuals [8].

Despite the overall lack of vaccine completion, the vaccination patterns demonstrated a clear relationship to vaccine requirements for school. In Pennsylvania, where the shelter is located, state law requires hepatitis B, DTaP, IPV, MMR, and varicella vaccinations for all school-aged children. Additionally, meningococcal and DTap vaccinations are required to enter seventh grade [9]. Our findings revealed that these immunizations all had >70% completion rates among homeless youth. Immunizations that are not required for schools, such as the Hib, hepatitis A, influenza, HPV, pneumococcal conjugate vaccine (PCV) 13, and rotavirus vaccines, demonstrated completion rates of <45%. The current literature has limited information on complete vaccination regimens among homeless youth in the United States [10,11]. However, our results mirror the data on vaccination coverage for school-required vaccines among all adolescents in the United States [12]. The adolescents in our study likely had adequate vaccination coverage for school-required vaccines because the majority of them attended school while they were at the shelter.

Albeit limited by missing data, we found a significant association between race and vaccine coverage, with less than one-quarter of Black/African American youth completing the CDC, recommended regimen. Previous studies on racial disparities have also reported correlations between race and immunization status [13]. Several studies found that black youth had significantly lower rates of influenza immunization compared to their white counterparts [14,15]. Interestingly, studies report conflicting data on comparative vaccination rates in Hispanics [13-15]. The adolescents included in our study already face great challenges due to housing instability and biopsychosocial disadvantages. This additional racial discrepancy further contributes to the obstacles that some of them have to overcome, in particular, an even higher risk of infection and illness compared to the already high risk among homeless individuals.

In a sample population where half of the individuals are sexually active, it is especially concerning that only 30.72% of the adolescents at the shelter completed their HPV vaccine regimens. According to the CDC, 51.1% of teens in a national survey reported completing the HPV series in 2018, with almost equal coverage rates among males and females [12]. Our findings revealed significantly more female homeless adolescents had completed the vaccine series than males, reflecting a gender disparity that has since diminished among national trends [12,16]. A possible explanation for the lack of vaccination completion among homeless adolescents is that they may be less likely to receive annual medical examinations, which have been shown to increase the odds of HPV immunization [17]. The gender differences seen in our population may be due to the lack of consistent medical care among homeless youth, along with the fact that the HPV vaccine was initially created for and promoted to females. Regardless, the homeless adolescent population is more likely to engage in high-risk sexual behaviors, and, as such, efforts should be made to improve HPV immunization rates among these individuals [18]. These findings suggest the need for further study into the reasons behind gender differences. Completing similar studies at other sites serving homeless youth may reveal geographical differences and shed additional light on immunization deficiencies and possible associations to specific biopsychosocial characteristics. Future studies may also include questions regarding sexual orientation to comment on how this may affect vaccination status. Additionally, future studies could prioritize capturing racial data more consistently.

Overall, further study into this population’s access to medical care providers, frequency of well-child checks, and health insurance status may explain what possible interventions might be most likely to improve vaccination status for adolescents experiencing homelessness.

Beyond investigating immunization status, our study collected important data on the biopsychosocial characteristics of homeless adolescents. These characteristics ultimately had no significant relationship to vaccination status, but still, highlight important issues that many homeless adolescents experience. We found that approximately one-quarter of our patients had experienced traumatic events, and more than half of our patients had a history of psychiatric conditions. This finding is not surprising as multiple studies show previous experiences of trauma are associated with higher occurrences of mental health issues among homeless youth [19,20]. Substance use and sexual activity were also common among our patients. These patterns in mental health, substance use, and sexual activity are potential targets for public health initiatives. By further study in the efficacy of programs to address these issues and behaviors among homeless youth, we can eventually close the gap on health disparities between them and their stably housed peers.

A major limitation of this study was the need to exclude many charts due to incomplete documentation. By nature of a retrospective study, the study was limited to the data that was collected in the medical record for clinical not research purposes. Immunization records were required to be available at the time of the patient encounter to be included in this study. If any of the vaccines were not given at the correct dosage or were missing from the electronic medical record (EMR) the vaccine regimen was considered incomplete for that specific record. From a public health perspective, this binary perspective may limit the interpretations that can be made from our results. Medical history, psychiatric history, behavioral history, social history, and sexual history were self-reported by patients or their parents, allowing recall bias. In addition, this study was conducted at a single site and may not account for geographical differences outside of our region, limiting generalizability. Furthermore, the length of stay at the shelter while generally short was not captured and may have influenced whether a child was due for vaccines during shelter stay. This is less likely due to the age of the clients and the fact that the influenza vaccine, the most likely to come due during a shelter stay, is offered by the clinic. Lastly, the examination indicated the patient’s gender identity but did not assess sexual orientation. The sample size of patients identifying as transgender was small. This limits full identification and assessment of the lesbian, gay, bisexual, transgender, and queer (LGBTQ) population and our ability to comment on how sexual orientation or gender identity may relate to vaccination status.

## Conclusions

The majority of adolescents experiencing homelessness in our study had the required doses of hepatitis B, MMR, IPV, and varicella vaccines. In contrast, less than half of these vulnerable youth had completed the appropriate doses for the Hib, hepatitis A, influenza, or HPV vaccine. Albeit limited by missing data, these findings were significantly influenced by race; less than one in five Black/African American patients had a completed vaccine regimen documented. Additionally, we found that a significantly greater number of female patients received the HPV vaccine compared to males. In general, this population seems to be under-vaccinated according to CDC recommendations, furthering their vulnerability for communicable and sexually transmitted infections. 
